# Indigenous Starter Cultures to Improve Quality of Artisanal Dry Fermented Sausages from Chaco (Argentina)

**DOI:** 10.1155/2015/931970

**Published:** 2015-01-31

**Authors:** Noelia Z. Palavecino Prpich, Marcela P. Castro, María E. Cayré, Oscar A. Garro, Graciela M. Vignolo

**Affiliations:** ^1^Laboratorio de Microbiología de Alimentos, Universidad Nacional del Chaco Austral, Comandante Fernández 755, Presidencia Roque Sáenz Peña, 3700 Chaco, Argentina; ^2^Consejo Nacional de Investigaciones Científicas y Técnicas (CONICET), C1033AAJ Buenos Aires, Argentina; ^3^Centro de Referencia para Lactobacilos (CERELA), Chacabuco 145, San Miguel de Tucumán, 4000 Tucumán, Argentina

## Abstract

Lactic acid bacteria (LAB) and coagulase negative cocci (CNC) were isolated from artisanal dry sausages sampled from the northeastern region of Chaco, Argentina. In order to evaluate their performance *in situ* and considering technological features of the isolated strains, two mixed selected autochthonous starter cultures (SAS) were designed: (i) SAS-1 (*Lactobacillus sakei* 487 + *Staphylococcus vitulinus* C2) and (ii) SAS-2 (*L. sakei* 442 + *S. xylosus* C8). Cultures were introduced into dry sausage manufacturing process at a local small-scale facility. Microbiological and physicochemical parameters were monitored throughout fermentation and ripening periods, while sensory attributes of the final products were evaluated by a trained panel. Lactic acid bacteria revealed their ability to colonize and adapt properly to the meat matrix, inhibiting the growth of spontaneous microflora and enhancing safety and hygienic profile of the products. Both SAS showed a beneficial effect on lipid oxidation and texture of the final products. *Staphylococcus vitulinus* C2, from SAS-1, promoted a better redness of the final product. Sensory profile revealed that SAS addition preserved typical sensory attributes. Introduction of these cultures could provide an additional tool to standardize manufacturing processes aiming to enhance safety and quality while keeping typical sensory attributes of regional dry fermented sausages.

## 1. Introduction

In Argentina, dry fermented meat products are manufactured by means of local artisanal techniques derived from Spanish and Italian traditions. Artisanal production lacks standardization processes, and relevant features—such as casing size, raw materials quality, and process parameters—are subjectively monitored [1]. Moreover, dry fermented meat products are produced without the addition of starter cultures; that is, the fermentation is conducted by wild-type strains, hence ensuring the development of the so-called indigenous—or autochthonous—microflora. Under these conditions, the settlement of the same group of microorganisms, giving a uniform fermentative behavior, is an improbable scenario. Consequently, the introduction of starter cultures in the manufacture of fermented meat products turns into a necessary implement in order to guarantee food safety and to standardize the final product attributes [2, 3].

Meat fermented products are the result of a complex microbiological activity that mainly consists of a lactic fermentation and several characteristic biochemical changes triggered by lactic fermentation. Meat starter cultures currently comprise coagulase negative cocci (CNC) and lactic acid bacteria (LAB). Many CNC species, such as* Staphylococcus* spp. and* Kocuria* spp., have nitrate reductase, proteolytic and lipolytic activities which contribute to the development of redness and flavor of the product [4–7]. As regards LAB, species mainly used as starter cultures are* Lactobacillus sakei*,* L. curvatus*,* L. plantarum*,* L. pentosus*,* L. casei*,* Pediococcus pentosaceus*, and* P. acidilactici* [8]. Lactic bacteria play a crucial role in this fermentation, that is, acidification, while they can also have proteolytic and lipolytic activities. The positive technological features of this acidification include (i) inhibition of spoilage and pathogenic microflora, (ii) quick drying and enhancement of texture due to denaturation and coagulation of proteins, (iii) activation of muscle proteases, and (iv) formation of nitric oxide and nitrosomyoglobin which contribute to redness [9]. However, an excessive acidification can negatively affect the taste or smell of the product and lead to color failures as a consequence of CNC inhibition [10]. The most important factors determining characteristics and quality of meat fermented products are the selection of starter cultures and the environmental conditions during fermentation and ripening [11, 12]. Quality of the products will be the result of the interaction between these two factors which generates a beneficial or a disadvantageous setting for cellular metabolism.

Indigenous microorganisms from meat fermentation are well adapted to fermentation and ripening conditions and are able to inhibit spontaneous growth of undesirable microflora [8]. Selected autochthonous starter cultures (SAS) can contribute to the manufacture of artisanal local products with particular features. In previous studies, two strains of* L. sakei*, one strain of* S. vitulinus*, and another of* S. xylosus* were isolated from regional meat fermented products [13]. Owing to their safety and technological features, these strains have potential application as starter cultures. In order to evaluate their performance* in situ* and, consequently, the feasibility of their use, these strains were tested as starter cultures in a local small-scale facility which manufactures dry fermented sausages. Two different mixed starter cultures were designed. Microbiological and physicochemical characteristics of the products had been monitored throughout the manufacturing process, while sensory attributes of the final products had been evaluated by a trained panel.

## 2. Materials and Methods

### 2.1. Autochthonous Starter Cultures

Strains used in this study were selected in previous studies taking into consideration their technological and safety features [13]; they are reported in Table 1. Association of the different strains to design each starter culture was determined by means of compatibility tests. Microorganisms are deposited in the Strain Repository of the Laboratorio de Microbiología de Alimentos (Universidad Nacional del Chaco Austral, Argentina). Lactobacilli are kept in MRS broth (de Man, Rogosa, Sharpe) supplemented with 20% v/v glycerol, while staphylococci are kept in soy trypticase + 0.6% w/v yeast extract (TSBYE) added with 20% v/v glycerol; both bacterial species are maintained frozen at −18°C.

Two mixed starter cultures were designed, namely, SAS-1 comprised of* L. sakei* 487 and* S. vitulinus* C2 and SAS-2 comprised of* L. sakei* 442 and* S. xylosus* C8. Lactic bacteria were propagated in MRS broth and CNC strains in TSBYE; they were incubated at 30°C for 12 h. Cells were then harvested by centrifugation (4500 rpm, 20 min), washed twice with saline peptone water (0.85 gNaCl/100 mL), and resuspended in this solution. Cellular density was monitored by plate count (MRS agar for LAB and Mannitol salt agar for CNC).

### 2.2. Dry Fermented Sausages Manufacture and Sampling

Dry fermented sausages used in this work were those currently manufactured by a local small-scale facility. Traditional recipes dictate the composition of these meat products per kilogram that consists of 450 g beef, 177 g pork meat, 300 g bacon, 34 g sodium chloride, 15 g sugar, 10 g powder milk, 0.7 g potassium nitrate, 3 g sodium glutamate, 5 g chopped garlic, 2 g ground red pepper, and 3 g ground black pepper. All the ingredients were thoroughly mixed together and meat dough was divided into two batches: one batch was added with the SAS and the other was kept as a control system. Afterwards, meat dough was packed and shaped into natural casings (sheep gut). SAS were added once the meat dough had been finished, being completely homogenized before casings were stuffed. Cultures SAS-1 and SAS-2 were added separately to the meat dough in two different instances. Final bacterial concentration (LAB + CNC) reached a value of ~10^6^ colony forming units per gram (cfu/g). After inoculation and stuffing, the unfermented products were placed in a ripening chamber for 15 days. During the first seven days—fermentation period—temperature was kept at 18–22°C and relative humidity (RH) oscillated to 90–95%. The next 8 days—ripening period—temperature was kept at 15°C and RH range was 80–85%. From each batch, samples were withdrawn to perform physicochemical and microbiological analyses at initial time, 2, 4, 7, and 15 days.

### 2.3. Microbiological Analyses

Ten grams of each sample was aseptically taken and was transferred to a stomacher bag with 90 mL of sterile peptone water to be homogenized. After 2 min in the stomacher (Stomacher 400 Circulator, Seward, UK), appropriate dilutions of the homogenate were done in order to inoculate several growth media to determine viable counts of LAB in MRS agar (30°C, 72 h); Micrococcaceae in AMS (30°C, 72 h); yeasts and molds in Chloramphenicol Sabouraud Agar (25°C, 5 days); Enterobacteriaceae in Glucose Red Violet Bile Agar (37°C, 24 h);* Staphylococcus aureus* in Baird Parker Agar with egg yolk emulsion and potassium tellurite (37°C, 48 h). Besides, final products were also assayed for the following:* Escherichia coli* count (MPN/g) following the ICMSF method [14]; sulphite-reducing anaerobes (cfu/g) according to ISO 15213:2003 [15]; absence of* E. coli* O157: H7/NM by the USDA-FSIS method [16]; absence of* Salmonella* spp. by the BAM-FDA method [17]; absence of* Listeria monocytogenes* according to ISO: 11290-1/A1 [18].

### 2.4. Physicochemical Analyses

#### 2.4.1. Water Activity (*a*_*w*_), pH, and Humidity

Water activity was measured at 25°C by means of a water activity device (Testo, Germany). Measurements of pH were determined with the aim of a pricking probe attached to a pH meter inserted directly into the meat dough (Testo). Humidity content (expressed as %) was determined according to the AOAC method [19]. These parameters were evaluated at every sampling stage.

#### 2.4.2. Ash, NaCl Concentration, and Fat Content

Ash content was analyzed according to the AOAC method [20]. NaCl concentration and fat content were determined according to AOAC [19]. These measurements were solely conducted on the final product.

#### 2.4.3. Lipid Oxidation Determination

Lipid oxidation was evaluated by means of the thiobarbituric acid reactive substances test (TBARS), according to the methodology suggested by Sinnhuber and Yu [21]. TBARS values were expressed as mg malonaldehyde per kilogram of sample (mg MDA/kg).

#### 2.4.4. Free Fatty Acid (FFA) Composition

The FFA compositions were determined in the fat fraction of dry sausage samples by means of gas chromatography of their corresponding methyl esters. Briefly, total lipid contents were extracted according to Bligh and Dyer [22], and then they were saponified, esterified, and transferred to n-heptane, as proposed by Hartman and Lago [23]. Fatty acid methyl esters were analyzed using an Agilent 6850AHP (Agilent Technologies Inc., CA, USA) chromatograph equipped with a capillary fused silica column Supelco 2340 (Supelco, Bellefonte, PA, USA) (60 m length, 0.25 mm bore, and 0.25 *μ*m film thickness), split–splitless injector, and flame ionization detector. The latter two were kept at a constant temperature of 250°C. Helium at 0.6 psi was the carrier gas. Temperature of the oven was set at 140°C for 5 min followed by an increase to 240°C at a rate of 4°C/min. The final temperature was kept constant for 20 min. FFA were identified by comparison of their retention times with those of authentic standards (Supelco 37 Components FAME Mixture, Bellefonte, PA). FFA concentration was expressed as mg fatty acid per gram of fat.

#### 2.4.5. Color Measurements

Color measurements were conducted in dry sausages cuts with the aim of an Evolution 600 UV-Vis spectrophotometer (Thermo Scientific, equipped with integrator sphere and VISION Lite Color Calc. Software, Germany), illuminant D65, and a 2° standard observer, in a room with fluorescent lighting and after standardization of the instrument with respect to the white calibration plate. CIE *L* ^*^, *a* ^*^, and *b* ^*^ values were determined as indicators of lightness, redness, and yellowness. Two sausages from each treatment were cut into 3 pieces. The color of each piece was measured at 5 locations on the covered cross section. The mean of 15 measurements was recorded for the color of the dry meat sausage.

### 2.5. Sensory Analysis

A quantitative descriptive analysis (QDA) was conducted by an eight-member sensory panel with previous experience in judging fermented sausages. Full ripened dry fermented sausages manufactured with the SAS and those used as control systems were evaluated. Two 30-minute-long training sessions were held—in which commercial fermented sausages were used—so as to define, discuss, and clarify each attribute to be evaluated. The overall assessment was performed following Baka et al. [2] guidelines. The evaluated attributes were as follows: (i) external appearance (descriptors: firmness, cohesiveness, and easy peeling capability of the case from the sausage); (ii) color (intensity and uniformity); (iii) aroma (intensity, cured smell, and rancidity); (iv) taste (salty, acid, and sour); (v) texture (chewing and hardening). The answer for every descriptor was determined as the mean value of panelists' answers.

### 2.6. Statistical Analyses

All data were expressed as means ± standard errors of duplicate measurements. Statistical comparisons between variables were performed with Student's *t*-test. Differences were considered significant at *P* < 0.05. One-way ANOVA was used to determine significant differences (*P* < 0.1) between sensorial attributes. All the tests were performed with the software Statgraphics Plus 4.0 (Manugistics Inc., USA).

## 3. Results and Discussion

### 3.1. Fermentative Bacteria and Associated Microflora

**Figure 1:**
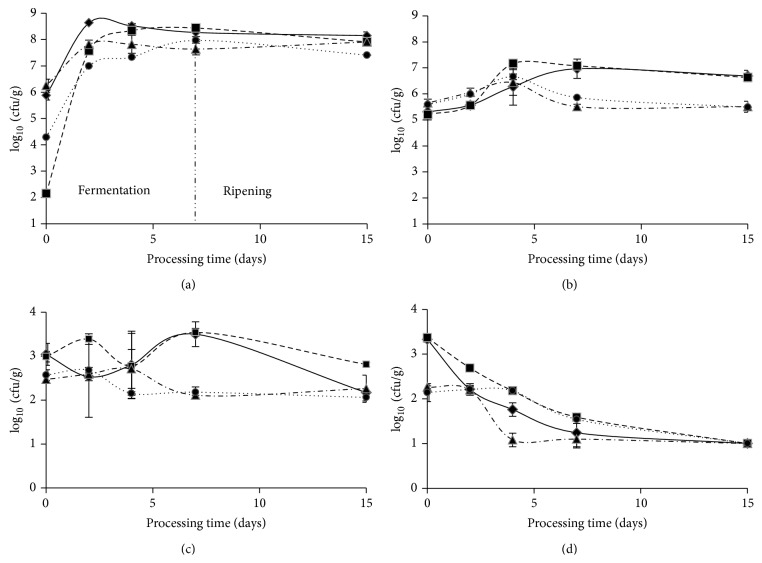
Bacterial counts of (a) lactic acid bacteria; (b) Micrococcaceae*;* (c) yeasts and molds; (d) Enterobacteriaceae, throughout fermentation and ripening periods. Systems: ◆-◆: SAS-1 (*Lactobacillus sakei* 487 +* Staphylococcus vitulinus* C2); ■—■: C-1 (noninoculated control system simultaneously manufactured with SAS-1); ▲- · -▲: SAS-2 (*L. sakei* 442 +* S. xylosus* C8); ●⋯●: C-2 (noninoculated control system simultaneously manufactured with SAS-2).

Lactic bacteria, molds and yeasts, Micrococcaceae, and Enterobacteriaceae counts are depicted in Figure 1.* S. aureus* was not detected in any of the samples taken during the productive process.

Lactic bacteria comprised the dominant microflora throughout fermentation and ripening periods. At the beginning of the productive process LAB counts in the inoculated systems were higher (SAS-1: 5.88 ± 0.18 log cfu/g and SAS-2: 6.26 ± 0.25 log cfu/g) than those of the control systems (C-1: 2.15 ± 0.01 log cfu/g and C-2: 4.29 ± 0.01 log cfu/g). Differences found in bacterial counts from both control systems reflected the high variability between batches that can be found in the processing plant. During the first 48 h, SAS-1 LAB counts rapidly increased to 8.64 ± 0.02 log cfu/g followed by a slight reduction to 8.27 ± 0.16 log cfu/g at the end of the fermentation period, with no significant variations until the end of the ripening period. As regards SAS-2, LAB counts reached a value of 7.82 ± 0.16 log cfu/g at 48 h and this value was kept invariable throughout the entire process. LAB counts from inoculated SAS-1 showed significant differences compared to the corresponding control system C-1 until the fourth day of fermentation whereas LAB counts from inoculated SAS-2 showed significant differences compared to its control system C-2 until the second day of fermentation. In addition, LAB counts of both control systems had been also increased during the fermentation period, reaching similar values compared to the inoculated systems at the end of the ripening period. The LAB imminent colonization in the systems inoculated with both SAS confirmed the good adaptation of indigenous LAB strains to the meat substrate, suggesting a high dominance and adaptability to the specific environmental conditions of the manufacturing process. Similar results had been reported by Baka et al. [2], Bonomo et al. [3], and Talon et al. [24], among others.

Micrococcaceae counts revealed no significant differences between inoculated systems (SAS-1 and SAS-2) and their respective control systems (C-1 and C-2) in any of the sampled stages. These results, which had also been reported by other authors [25, 26], can be explained in the view that a great load of this microbial group could inhabit the processing environment facilitating its introduction into the meat dough during mixing and handling. Nevertheless, Micrococcaceae are weak competitors for nutrients compared to LAB, though their counts were not higher than LAB's counts. Regarding molds and yeasts counts, they did not show significant differences between inoculated systems and their noninoculated control systems during the studied period. Enterobacteriaceae showed bacterial counts that did not differ significantly between SAS-1 and C-1 nor between SAS-2 and C-2 (mean values were 3.35 ± 0.06 and 2.20 ± 0.18 log cfu/g, resp.). Similar counts for this microbial group were encountered in meat fermented products from other regions [2, 3, 27]. Bacterial counts from this group diminished significantly at the second day of fermentation in system SAS-1, while this reduction was evident for SAS-2 only after the fourth day. However, in the different systems (inoculated and noninoculated) bacterial counts were less than 1 log cfu/g at the end of the ripening period (Figure 1(d)). The latter behavior is expected since Enterobacteriaceae are highly sensitive to acidity and dryness [28]. In general, Gram-negative bacteria and Enterobacteriaceae are considered unwanted microflora in meat fermented products, the dominance of LAB and Micrococcaceae being an essential event on the manufacture of these products. Besides, quality control of the final products showed that all the samples were within the desirable values for good hygiene and food safety of this type of products, namely,* E. coli* (MPN/g) < 3; sulphite-reducing anaerobes < 10 cfu/g;* Salmonella* spp. absence in 25 g of sample;* Listeria monocytogenes* absence in 25 g of sample; and* E. coli* O157: H7 absence in 65 g of sampled products.

### 3.2. Physicochemical Parameters

**Figure 2:**
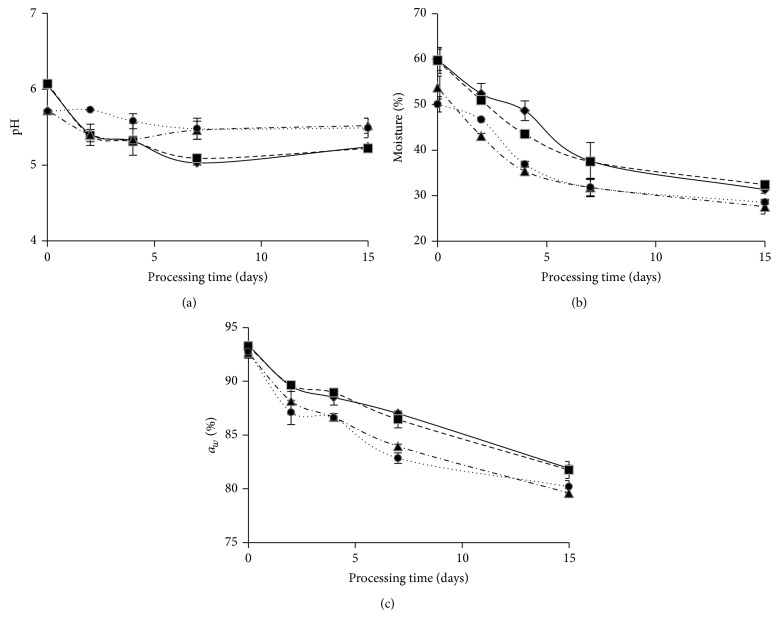
Physicochemical parameters determined during dry sausages fermentation and ripening periods. (a) pH values; (b) humidity (%); (c) *a*_*w*_ (%). Systems: ◆-◆: SAS-1 (*Lactobacillus sakei* 487 +* Staphylococcus vitulinus* C2); ■—■: C-1 (noninoculated control system simultaneously manufactured with SAS-1); ▲- · -▲: SAS-2 (*L. sakei* 442 +* S. xylosus* C8); ●⋯●: C-2 (noninoculated control system simultaneously manufactured with SAS-2).

The results of pH, *a*_*w*_, and humidity measurements during fermentation and ripening of dry sausages are reported in Figure 2. Initial pH values in the meat matrix were not significantly different between SAS-1 and C-1 nor SAS-2 and C-2 (mean values 6.06 and 5.72, resp.). Furthermore, no significant differences were found between pH values of SAS-1 systems and their control systems throughout the productive process. Values for SAS-2 showed a significant reduction after two days of fermentation compared to the control system (5.4 and 5.7, resp.); however, this difference disappeared on subsequent days; pH values became similar (~5.47) at the end of the fermentation period. Comparing SAS-1 to SAS-2, the first contributed to a deeper acidification of the fermented system; that is, SAS-1 produced a pH falling of 0.81 units, while SAS-2 just decreased pH in 0.2 units. This reduction is due to carbohydrate fermentation within the meat matrix mediated by LAB which produces organic acids responsible for pH decrease. This situation in meat fermentation is crucial since it contributes to the inhibition of undesirable microflora, accelerates the development of redness, affects flavor, and reduces water holding capacity of proteins contributing to the necessary drying process [29]. A slight pH increase in all the systems (with the exception of system C-2) was recorded during the ripening process; it can be attributed to the deamination effect caused by yeasts [30, 31] and to the buffering capacity of proteins [32]. In general, pH values found herein are in agreement with those found by other authors [3, 26, 27].

Humidity values at the end of the ripening period ranged between 27.58 and 32.41% in all the samples (inoculated and noninoculated). No significant differences were found between SAS and control systems humidity values in any of the different stages of the productive process. Similar results were reported by Palavecino et al. [33] and Romero et al. [34] for products manufactured in the same geographical region, as well as Casaburi et al. [27] and Casquete et al. [35] for European products. Nevertheless, these values were considerably lower than others reported for products from other regions where for longer ripening periods humidity contents were higher [3, 7, 12, 36, 37].

*a*_*w*_  showed high values at the beginning of the process (92.65–93.35%) and then it diminished progressively to final values ranging between 79.6 and 81.9% for all the systems. No significant differences were found between inoculated systems and their respective controls.

Other physicochemical parameters evaluated on final products are presented in Table 2. As regards ash content, NaCl concentration, and lipid content, no significant differences were determined between inoculated and noninoculated systems. On the contrary, TBARS values were higher in control systems C-1 and C-2 compared to systems SAS-1 and SAS-2, respectively. Natural casings used to stuff dry sausages are permeable to air, and, consequently, sausages are exposed to lipid oxidation. TBARS values were significantly higher in control system C-2 and from these results it could be inferred that SAS are able to prevent lipid oxidation in dry meat sausages and they restricted TBARS values to a concentration of 1 mg MDA/kg, which is considered the rancidity limit for fresh meat [38]. Differences found amongst SAS could be attributed to their different antioxidant properties, namely, metal chelating capacity, free radical scavenging, and reducing properties. Although TBARS values close to 2 mg MDA/kg had been found in fermented meat products [39, 40] and even higher amounts (2.2 mg MDA/kg), they were not enough to be detected as rancid at a sensory level [41].

Changes in color parameter are shown in Table 3. Results showed that there were significant differences in the redness in those samples inoculated with SAS-1 compared to their control systems (C-1). Redness in fermented meat products is due to nitrosomyoglobin formation when nitrogen monoxide reacts with myoglobin. Nitrosomyoglobin increases exponentially at the beginning of the drying process as a result of nitrate reductase activity from staphylococci [42]. Moreover, Micrococcaceae have the ability to produce the enzyme catalase which destroys the oxygen peroxide produced by LAB. Oxygen peroxide affects heme pigments and can produce oxidative decoloration of dry fermented sausages. From these results it could be inferred that the nitrate reductase positive strain* S. vitulinus* C2 (part of SAS-1) was effective in achieving a better redness development with the aim of the lower pH found in these samples compared to the SAS-2 ones. Regarding the rest of the color parameters, no significant differences were found between the inoculated systems and their control systems. On account of fat content, hematic pigments, nitrite/nitrate concentration, and humidity, among other factors, which affect reflectance values from these products, different chromatic values had been reported by many authors [43–45].

### 3.3. FFA Profile

Free fatty acids concentration (expressed as mg/g of fat) was determined in order to evaluate SAS effect on lipid fraction of studied meat products. Samples manufactured with the addition of SAS showed a similar lipidic profile to the noninoculated samples. These results are shown in Table 4. Talon et al. [24] and Casaburi et al. [27] described similar tendencies. Significant differences for oleic acid concentration were found in system SAS-1, whose strains did not show lipolytic activity* in vitro*, compared to its control system C-1. This fact could be attributed to endogenous lipases activity, being favored by the pH decrease [46]. On the other hand, lipolytic strain* S. xylosus* C8 (part of SAS-2) was not effective in the lipolysis of the products during ripening, though endogenous lipases activity can be confirmed [47, 48]. Free fatty acids—released as a consequence of endogenous lipases and microbial activity—comprise the substrate for those biochemical reactions that lead to aromatic compounds formation and flavor of the final product [3, 49, 50].

### 3.4. Sensory Attributes

**Figure 3:**
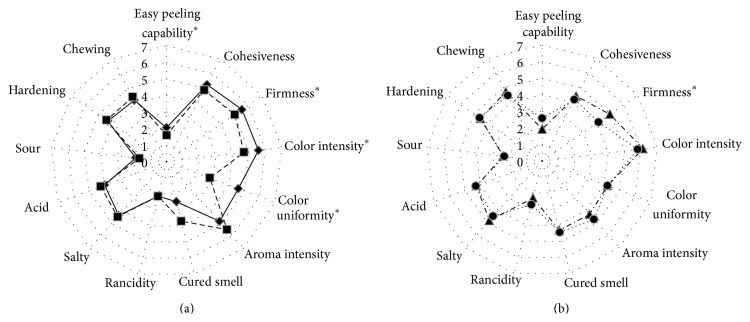
Effect of autochthonous starter cultures on the sensory attributes of fermented sausages. (a) Comparison between SAS-1 and its control system C-1. (b) Comparison between SAS-2 and its control system C-2. Systems: ◆-◆: SAS-1 (*Lactobacillus sakei* 487 +* Staphylococcus vitulinus* C2); ■—■: C-1 (noninoculated control system simultaneously manufactured with SAS-1); ▲- · -▲: SAS-2 (*L. sakei* 442 +* S. xylosus* C8); ●⋯●: C-2 (noninoculated control system simultaneously manufactured with SAS-2). ^*^Significantly different (*P* < 0.1).

Sensory quality of dry meat sausages (texture, color, and flavor) depends on numerous compounds involved in many biochemical reactions that take place during the manufacturing process. Final firmness of the products is the result of its acidification and dryness. Both events are favored by lactic acid bacteria owing to their acidifying capacity which also contributes to the dryness of the product because it reduces the water holding capacity of the mixture [51]. In this study, an enhancement in the firmness of inoculated dry sausages was observed, which can be attributed to the* L. sakei* strains acidification activity from both SAS. Samples inoculated with SAS-1 (Figure 3(a)) also showed higher punctuation for the following descriptors: easy peeling capability of the case from the sausage, color intensity, and uniformity. The higher color intensity is in keeping with the higher redness found in those samples inoculated with SAS-1. On the other hand, samples inoculated with SAS-2 (Figure 3(b)) obtained lower scores for the descriptor easy peeling capability of the case from the sausage. No significant differences in the rest of the evaluated attributes were observed compared to the control systems, confirming that the designed SAS could contribute to enhance the quality of these traditional local products without changing their typical sensory attributes.

## 4. Conclusions

Native starter cultures represent an efficient alternative to commercial starter cultures since the latter do not always offer the desirable properties to the final product. Selected autochthonous starters enhanced safety and hygienic profile of dry sausages, showing also a beneficial effect on lipid oxidation and texture of the final products. Sensory profile revealed that SAS addition helped to preserve typical sensory attributes inasmuch as any of the SAS affected it significantly. Finally, the addition of these cultures could provide an additional tool to standardize manufacturing processes aiming to enhance safety and quality while keeping typical sensory attributes of regional dry fermented sausages. Further studies are being conducted in order to test the robustness of both SAS in different production batches throughout the year.

## Figures and Tables

**Table 1:** Indigenous bacterial strains used as starter cultures in this study.

Strain	Technological activity
*Lactobacillus sakei* 442	Acidifying, proteolytic, and nitrate reductase
*Lactobacillus sakei* 487	Acidifying, proteolytic, and nitrate reductase
*Staphylococcus vitulinus* C2	Proteolytic and nitrate reductase
*Staphylococcus xylosus* C8	Lipolytic and nitrate reductase

**Table 2:** Ash and NaCl concentration, total fat content, and TBARS values registered for dry sausages at the end of the ripening period.

System	Ash (%)	NaCl (%)	Total fat (%)	TBARS (mg MDA/kg)
SAS-1	6.9^a^ ± 0.31^b^	6.11 ± 0.97	24.97 ± 4.29	0.89 ± 0.03
C-1	6.42 ± 0.15	5.39 ± 0.38	21.46 ± 2.67	1.05 ± 0.08
SAS-2	6.95 ± 0.32	5.73 ± 0.18	30.05 ± 2.62	0.97 ± 0.04
C-2	6.38 ± 1.12	6.03 ± 0.01	33.99 ± 0.55	1.54 ± 0.04

^a^Mean value.

^b^Standard deviation.

**Table 3:** Color determination in dry sausages at the end of the ripening period.

System	*L* ^*^	*a* ^*^	*b* ^*^
SAS-1	40.67^a^ ± 0.89^b^	9.99 ± 0.66	5.86 ± 0.37
C-1	41.35 ± 0.57	9.10 ± 0.55	5.55 ± 0.53
SAS-2	39.04 ± 1.05	8.53 ± 1.08	5.38 ± 0.62
C-2	38.68 ± 2.36	8.36 ± 0.76	4.90 ± 0.27

^a^Mean value.

^b^Standard deviation.

**Table 4:** Free fatty acid (FFA) profile [mg fatty acid/g of fat] of dry sausages manufactured with indigenous starter cultures.

Free fatty acid	System			
	SAS-1	C-1	SAS-2	C-2
Saturated				
(14:0), myristic	10.93^a^ ± 0.99^b^	9.55 ± 0.84	11.99 ± 3.01	11.38 ± 2.15
(16:0), palmitic	178.16 ± 13.9	152.75 ± 16.3	189.85 ± 9.08	178.91 ± 7.75
(17:0); heptadecanoic	3.29 ± 0.43	2.96 ± 0.49	2.66 ± 0.57	2.57 ± 0.44
(18:0), stearic	92.25 ± 6.58	76.05 ± 6.43	87.49 ± 20.1	18.14 ± 21.8
(22:0), behenic	8.32 ± 0.70	7.30 ± 2.1	5.60 ± 1.64	5.07 ± 1.60
Monounsaturated				
(16:1), palmitoleic	17.93 ± 1.46	15.51 ± 1.65	18.51 ± 3.31	17.78 ± 2.28
(17:1)c, heptadecanoic	3.14 ± 0.35	2.57 ± 0.47	2.42 ± 0.42	2.37 ± 0.20
(18:1)c; n9 oleic	331.87 ± 5.92	257.06 ± 4.37	301.02 ± 13.9	305.52 ± 10.3
Polyunsaturated				
(18:2)c, n6 linoleic	79.41 ± 4.57	69.07 ± 10.05	74.35 ± 12.17	73.36 ± 10.53
*α*(18:3) n3, heneicosanoic	9.98 ± 0.68	8.97 ± 0.65	8.17 ± 1.64	7.65 ± 1.57
(20:3)c, n3 1,14,17-eicosatrienoic	1.28 ± 0.26	1.29 ± 0.25	ND	ND

^a^Mean value.

^b^Standard deviation.

ND: non detected.
